# Secreted Frizzled-Related Protein Promotes Bone Regeneration by Human Bone Marrow-Derived Mesenchymal Stem Cells

**DOI:** 10.3390/ijms161023250

**Published:** 2015-09-25

**Authors:** Wataru Katagiri, Masashi Osugi, Takamasa Kawai, Hideharu Hibi

**Affiliations:** Department of Oral and Maxillofacial Surgery, Nagoya University Graduate School of Medicine, 65 Tsuruma-cho, Showa-ku, Nagoya 466-8550, Japan; E-Mails: masashi@med.nagoya-u.ac.jp (M.O.); takamacha1@hotmail.com (T.K.); hibihi@med.nagoya-u.ac.jp (H.H.)

**Keywords:** human mesenchymal stem cells (hMSCs), secreted frizzled-related protein-3 (sFRP-3), Wnt signaling, osteogenic differentiation, bone regeneration

## Abstract

Secreted frizzled-related protein (sFRP)-3 is a negative regulator of Wnt signaling in human mesenchymal stem cells (hMSCs). The present study investigated the effects sFRP-3 on osteogenic differentiation by assessing osteogenic gene expression in hMSCs *in vitro* and by examining bone regeneration in a rat bone defect model. sFRP-3 treatment induced osteogenic differentiation in hMSCs as determined by *alkaline phosphatase*, *collagen type I*, *osteocalcin*, and *Runt-related transcription factor 2* gene expression. hMSCs with or without sFRP-3 were implanted into a rat calvarial bone defect; a radiographic analysis by micro-computed tomography and histological analysis 4 and 8 weeks after implantation showed greater bone regeneration in the sFRP(+) than in the sFRP(−) group. These results suggest that modulation of Wnt signaling contributes to osteogenic differentiation in hMSCs. Specifically, sFRP-3 induces osteoblastic differentiation of cultured MSCs and bone regeneration in a calvarial bone defect, suggesting that it can be a useful agent for the treatment of bone defects.

## 1. Introduction

Until recently, autogenous bone grafts were considered the gold standard for the treatment of bone defects resulting from surgery, trauma, and inflammatory disease. This well-studied procedure has good prognosis but requires an extra surgery at the donor site, which is an additional burden on the patient. Although several bone substitutes are commercially available, these materials tend to be easily infected and induce antigenic responses. An alternative treatment strategy for bone defects is growth factor therapy; factors such as bone morphogenetic protein 2 have demonstrated excellent osteogenic potential [1,2] and are already available on the market. However, high physiological doses are required to produce an effect [3] and a severe inflammatory response may be induced [4,5], while the amount of bone that can be regenerated is limited. Therefore, there is a need to establish novel bone regeneration techniques that circumvent these issues.

Tissue engineering involves the generation of new tissue from isolated cells with scaffolds and growth factors [6]. While pluripotent stem cells such as embryonic stem cells and induced pluripotent stem cells have a greater potential for regenerative medicine, adult stem cells are currently more widely used for therapeutic applications owing to ethical and safety considerations. Among the various types of adult stem cell, bone marrow-derived mesenchymal stem cells (MSCs) have been extensively studied and used in various therapeutic applications. We previously used a mixture of human (h)MSCs and platelet-rich plasma (PRP) (hMSCs/PRP)—bone graft materials with predictable grafting success—for craniofacial reconstruction and dental implants [7], and bone regeneration using hMSCs/PRP has been successful in some clinical settings [8]. hMSCs can be obtained by a minimally invasive technique and their use can obviate the need for donor site surgery in autogenous bone graft procedures. However, there are problems associated with hMSC-based tissue regeneration, such as the variability in terms of cell proliferation and differentiation time among patients. A more effective osteogenic induction method that reduces culture time while enhancing bone quantity and quality is desired.

Wnts constitute a large family of secreted signaling molecules that play a number of important roles during animal development [9]. The canonical Wnt signaling pathway regulates cytoplasmic accumulation of β-catenin, leading to the downstream transcription of Wnt target genes [10]. Canonical Wnt signaling is known to be involved in the self-renewal of hematopoietic stem cells [11] and has been implicated in bone formation; for instance, osteoporosis pseudoglioma syndrome is associated with an inactivating mutation in the Wnt co-receptor low-density lipoprotein receptor-related protein 5 [12,13], providing evidence that canonical Wnt signaling is a key regulator of osteogenesis.

Wnt signals are also important for hMSC proliferation and differentiation. Wnt 3a, a canonical Wnt member, induces β-catenin nuclear localization and MSC proliferation while inhibiting osteogenic differentiation. Low concentrations of the Wnt mimic lithium, which inhibits phosphorylation of β-catenin by GSK-3β, also stimulates hMSC proliferation [14,15,16]. On the other hand, the Wnt inhibitor secreted Frizzled-related protein (sFRP)-3 is upregulated in MSC osteogenesis [14]. sFRP-3 was shown to induce osteoblastic differentiation of hMSCs by binding Wnt5A and thereby antagonizing non-canonical Wnt signaling [17]. The modulation of Wnt signaling by sFRP-3 has therapeutic implications for the treatment of bone defects, since it may accelerate regeneration and thus shorten the treatment period and reduce the cost of cell culture. However, little is known about the *in vivo* effects of sFRP-3-treated hMSCs on bone regeneration. The present study investigated the role of sFRP-3 in the osteogenic potential of hMSCs *in vitro* and on bone regeneration *in vivo* in a rat calvarial bone defect model.

## 2. Results and Discussion

### 2.1. sFRP-3 Induces the Expression of Osteogenic Markers in hMSCs

**Figure 1:**
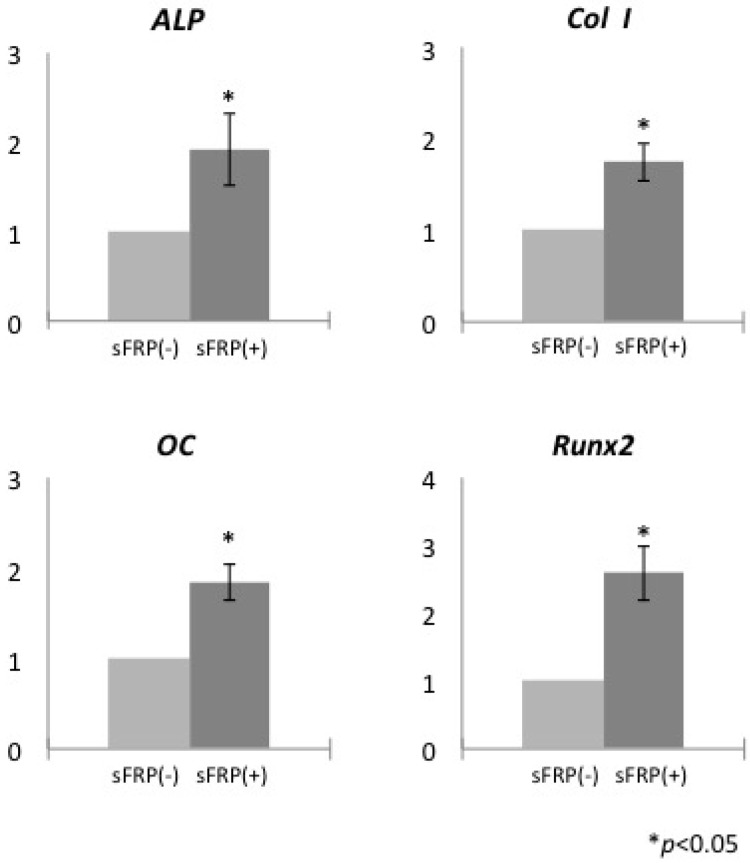
Relative mRNA expression of osteogenesis-related genes in human mesenchymal stem cells (hMSCs) cultured with or without secreted frizzled-related protein (sFRP-3). * *p* < 0.05.

The expression of *ALP*, *collagen type I* (*ColI*), *osteocalcin* (*OC*), and *Runt-related transcription factor* (*Runx*)*2* was assessed relative to *glyceraldehyde 3-phosphate dehydrogenase* (*GAPDH*) by quantitative real-time (qRT-)PCR on day 14 in hMSCs cultured with or without sFRP-3. The mRNA levels of each of these genes associated with osteogenic differentiation were upregulated in hMSCs treated with sFRP-3 as compared to untreated cells (Figure 1).

sFRP-3 belongs to a family of soluble proteins that are structurally related to Frizzled Wnt receptors; indeed, they are considered as decoy receptors that bind to Wnt proteins to prevent signal activation [18]. sFRP-1 inhibits Wnt-β-catenin signaling in osteoblasts, resulting in the loss of bone mass in adult mice, and sFRP-1-null mice exhibit increased trabecular bone mineral density, volume, and mineral apposition rate as compared to controls [19]. Human multiple myeloma cells constitutively produce sFRP-2 and suppress bone formation at least in part via inhibition of Wnt signaling [20]. sFRP-3 and -4 exhibit opposite effects on osteoblastic differentiation of hMSCs: the former promotes whereas the latter suppresses this process [21]. Thus, sFRP is critical for bone formation and acts primarily by antagonizing Wnt signaling.

The effects of Wnt signaling differ according to cell background and origin. For example, ALP was activated in C3H/10T1/2 cells from mouse embryos [22] but inactivated in hMSCs [14,15] by Wnt signaling. Thus, the effects of Wnt signaling on cellular proliferation and differentiation are cell type-specific although the mechanistic basis for these differences is poorly understood. It was previously demonstrated that *ALP* and *bone sialoprotein* expression was downregulated in hMSCs induced with Wnt3a but upregulated in the presence of both Wnt3a and sFRP-3 [14]. In addition, transfection of a short interfering RNA against sFRP-3 suppressed ALP activity [21]. These findings along with the present results indicate that Wnt signaling negatively regulates while sFRP-3 activates osteogenic differentiation in hMSCs. In this study, sFRP-3 was used for accelerating osteogenic differentiation of hMSCs by inhibiting Wnt-β-catenin pathway. We found 10 ng/mL of sFRP-3 had an inhibitory effect on the cellular proliferation by bromodeoxyuridine (BrdU) staining assay (Figure S1). Furthermore, this inhibitory effect on cellular proliferation was neutralized by the addition of Wnt mimic lithium chloride (LiCl) (Figure S2). Other study showed that Wnt mimic LiCl (4 mM) inhibited osteogenic differentiation of hMSCs [15] and our preliminary study also indicated that the addition of 4 mM of LiCl accelerated the growth rate of hMSCs (data not shown). Immunocytochemistry also revealed the effects of sFRP-3 and LiCl on Wnt-β-catenin pathway in hMSCs. The addition of LiCl enhanced the localization of β-catenin in the nucleus, whereas the addition of sFRP-3 inhibited the localization of β-catenin in the nucleus (Figure S3). Thus, sFRP-3 regulated Wnt-β-catenin pathway negatively and LiCl regulated positively in hMSCs. We also found that calcification and ALP activity are increased in hMSCs upon sFRP-3 treatment (Figure S4), as the previous study reported [21].

Osteogenesis-related genes are targets of Wnt signaling; this has been demonstrated by the identification of a binding site for T cell factor—a transcriptional co-activator of canonical Wnt signaling—in the promoter of the *Runx2* gene that functions in bone formation [23]. We found that the expression of *ALP*, *ColI*, *OC*, and *Runx2* was upregulated in sFRP-3-treated cells on day 14, providing evidence that inhibition of Wnt signaling by sFRP-3 promotes osteogenic differentiation of hMSCs.

### 2.2. sFRP-3 Enhances Bone Regeneration in Vivo

**Figure 2:**
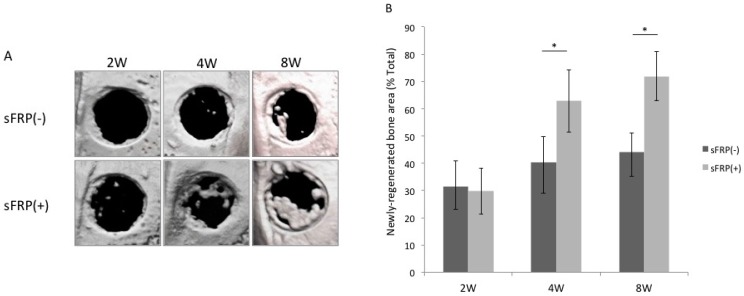
Micro-computed tomography (Micro-CT) analysis of bone regeneration in a rat calvarial bone defect model. (**A**) Micro-CT images of the bone 2, 4 and 8 weeks after transplantation of hMSCs/Terudermis or hMSCs/sFRP-3/Terudermis (sFRP(−) and (+), respectively); (**B**) Area of newly regenerated bone (mm^2^) in each defect 2, 4 and 8 weeks after transplantation expressed as a percentage of the entire defect area. * *p* < 0.05.

To determine whether sFRP-3 can induce osteogenic differentiation of hMSCs *in vivo*, we transplanted hMSCs/Terudermis or hMSCs/sFRP-3/Terudermis (sFRP(−) and (+), respectively) into rat calvarial bone defects and measured the area of newly regenerated bone as a percentage of the total graft area at 2, 4 and 8 weeks post-transplantation by micro-computed tomography (micro-CT) (Figure 2A). The area of newly regenerated bone was greater in the sFRP(+) than in the sFRP(−) group at 4 weeks (62.9% ± 6.9% *vs.* 40.5% ± 9.3%) and at 8 weeks (71.9% ± 8.9% *vs.* 44.1% ± 11.5%), although there was no significant difference at 2 weeks (29.7% ± 8.4% *vs.* 31.5% ± 9.4%) (Figure 2B).

**Figure 3:**
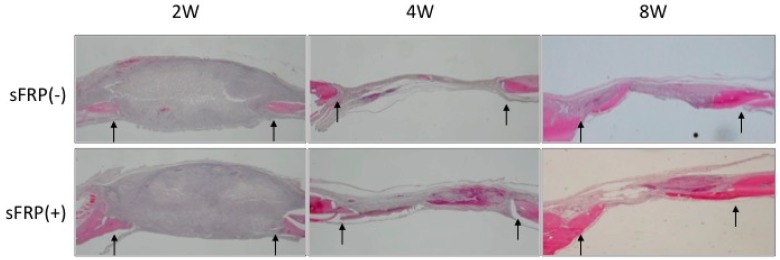
Histological evaluation of newly regenerated calvarial bone in rats. hMSCs with or without sFRP treatment were transplanted into rats and tissue sections were examined 2, 4 and 8 weeks later by staining with hematoxylin and eosin (12.5×). Arrows indicate the edges of the bone defect.

A histological analysis revealed that bone was regenerated to a greater degree in the sFRP(+) as compared to the sFRP(−) group at 4 and 8 weeks. At 4 weeks, the bone defect was partially covered with newly regenerated bone in the sFRP(+) group and with connective tissue in the sFRP(−) group. At 8 weeks, there was some newly regenerated bone detected within the defect in the sFRP(−) group, but the defect was filled with mature bone tissue in the sFRP(+) group. At 2 weeks, post surgical swelling was seen in each specimen with little bone tissue regeneration (Figure 3). These findings from micro-CT and histological analysis indicated that hMSCs with or without sFRP-3 treatment themselves survived keeping their cellular osteogenic characteristics and mineralized after removal of inflammatory tissue observed at 2 weeks.

The stimulation of bone regeneration by sFRP-3 suggests that Wnt signaling normally inhibits osteogenic differentiation *in vitro* and bone regeneration *in vivo*. The inclusion of sFRP-3 in osteogenic cultures of hMSCs can therefore shorten culture time and promote early bone regeneration in clinical applications.

## 3. Experimental Section

### 3.1. Cell Culture

Commercially available human primary marrow-derived MSCs (Lonza, Walkersville, MD, USA) were cultured at 37 °C in a 5% CO_2_ atmosphere in MSCGM consisting of MSC basal medium and SingleQuots mesenchymal cell growth supplement, l-glutamine, and penicillin/streptomycin (Lonza). The hMSC osteogenic differentiation Bullet kit (Lonza) was used for osteogenic induction, which contained dexamethasone, ascorbate, mesenchymal cell growth supplement, l-glutamine, penicillin/streptomycin, and β-glycerophosphate. The medium was refreshed every 3 days. Recombinant human sFRP-3 (R & D Systems, Minneapolis, MN, USA) was used at a concentration of 10 ng/mL in the osteogenic induction medium.

### 3.2. qRT-PCR

Reverse transcription was performed using TaqMan EZ RT-PCR Core Reagents (Applied Biosystems, Foster City, CA, USA) according to manufacturer’s instructions with 100 ng of RNA. qRT-PCR was performed on an ABI PRISM 7000 Sequence Detector System (Applied Biosystems). Specific primers and probes are listed in Table 1. *GAPDH* primer and probe (TaqMan GAPDH detection reagents) were purchased from Perkin-Elmer (Waltham, MA, USA) and Applied Biosystems. Cycling conditions were 2 min at 50 °C, 30 min at 60 °C, 5 min at 95 °C, and 50 cycles of 20 s at 95 °C and 1 min at 60 °C. Relative mRNA expression levels were normalized to that of *GAPDH* and data from samples with sFRP-3 treatment were also normalized to those from samples without sFRP-3 treatment.

**Table 1:** Specific primer and probe sequences used for qRT-PCR.

Gene		Sequence	Accession No.
*ALP*	F	AGAAAGCCAGGGGCACGAG	NM_000478
	R	GGGAGTGCTTGTATCTCGGTTTG	
	Probe	CCTGGACCTCGTTGACACCTGGAAGAGC	
*ColI*	F	GACAGTCATTGAATACAAAAC	NM_053356
	R	ACGGAATTCTTGGTTAGTA	
	Probe	TAAGCCATCTCGCCTGCCAT	
*OC*	F	GACTCTGAGTCTGACAAA	NM_013414
	R	AGTCCATTGTTGAGGTAG	
	Probe	CATCCATCCATTCCACCACGC	
*Runx2*	F	CCTCTTATCTGAGCCAGA	NM_053470
	R	GCAGTGTCATCATCTGAA	
	Probe	CATCCATCCATTCCACCACGC	
*GAPDH*	F	GTTCCAGTATGACTCTACC	NM_017008
	R	TCACCCCATTTGATGTTA	
	Probe	TTCAACGGCACAGTCAAGGC	

F: forward; R: reverse.

### 3.3. Rat Calvarial Bone Defect Model

**Figure 4:**
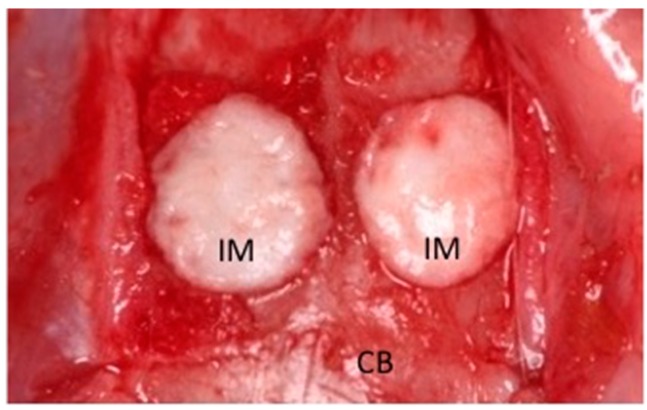
Experimental materials implanted into a rat calvarial bone defect. CB, Calvarial bone; IM, Implanted material.

Animal experiments were performed in strict accordance with protocols approved by the Animal Care Committee of Nagoya University Graduate School of Medicine (approval No. 23385 and 23414). Male Wistar/ST rats (10 weeks old; *n* = 8) were anesthetized by intraperitoneal injection of pentobarbital (Somnopentyl; Kyoritsu Seiyaku, Tokyo, Japan) (20 mg/kg body weight). Two circular full-thickness bone defects (5 mm in diameter) were made in the calvarial bone using a trephine bur and were irrigated with saline to remove bone debris. The experimental materials were then transplanted into the defects (Figure 4). Atelocollagen (Terudermis; Olympus Terumo Biomaterials, Tokyo, Japan), which was cut into the desired form, was resuspended with hMSCs (with or without sFRP-3 treatment; 1 × 10^5^/defect) in PBS. We defined the following groups based on the implanted materials: sFRP(−), hMSCs/Terudermis and sFRP(+), hMSCs/sFRP-3/Terudermis. Rats were sacrificed at 2, 4 or 8 weeks after transplantation (*n* = 5 per group).

### 3.4. Radiographic and Histological Analysis

Surgical sites were dissected, fixed in 4% paraformaldehyde, and subjected to micro-CT analysis using the R_mCT system (Rigaku, Tokyo, Japan). The X-ray source operated at 90 kV and 88 μA, scan time 2 min, image range φ 24 mm × 24 mm and voxel size 50 μm × 50 μm × 50 μm. The X-ray tube and flat panel rotate around the specimen to take cross-sectional X-ray CT images. The images were reconstructed on a personal computer using I-VIEW software (J. Morita, Kyoto, Japan) and analyzed using TRI/3D-BON 64 software (RATOC, Tokyo, Japan). After radiological assessment, explants were decalcified with K-CX solution (Falma, Tokyo, Japan) and dehydrated in a graded series of ethanol, cleared with xylene, and embedded in paraffin. Specimens were cut sagittally to obtain 3-μm-thick histological sections that were stained with hematoxylin and eosin.

### 3.5. Statistical Analysis

All experiments were conducted in triplicate and repeated at least twice. Group means and standard deviations were calculated for each measured parameter. Statistical differences were evaluated with Tukey’s honestly significant difference test. *p* < 0.05 was considered statistically significant.

## 4. Conclusions

sFRP-3 was shown to regulate osteogenic differentiation of hMSCs and promote bone regeneration in a rat calvarial bone defect. These findings suggest that sFRP-3 can be novel therapeutic agent for bone regeneration.

## Supplementary Material

Supplementary materials can be found at http://www.mdpi.com/1422-0067/16/10/23250/s1.
