# Supplementary material for: Secreted Frizzled-Related Protein Promotes Bone Regeneration by Human Bone Marrow-Derived Mesenchymal Stem Cells
Source: Int J Mol Sci. 2015 Sep 25;16(10):23250–8. doi: 10.3390/ijms161023250 (PMC4632696; doi:10.3390/ijms161023250)
Supplement: Supplementary file 1 [file ijms-16-23250-s001.pdf]

Supplementary Information

1. Bromodeoxyuridine (BrdU) Assay

The proliferation rate of 70%–80% confluent hMSCs was assessed by bromodeoxyuridine incorporation for 24 h, using a Zymed BrdU staining kit (Invitrogen, Carlsbad, CA, USA), according to the manufacturer's instructions. The number of BrdU-positive cells was counted using a light microscope.

2. Immunofluorescent Antibody Staining

Cells grown on cover slips were fixed with PBS containing 4% paraformaldehyde for 15 min and then rendered permeable with PBS containing 0.1% Triton X-100 for 3 min at 4 °C. After blocking with 2% bovine serum albumin in PBS for 30 min, the cells were incubated with anti-β-catenin mouse monoclonal antibody (BD Biosciences, San Jose, CA, USA) diluted 1:500 in blocking solution at 4 °C overnight. Then, the cells were washed and incubated with a 1:150 dilution of goat anti-mouse secondary antibody conjugated to rhodamine (Leinco Technologies, St. Louis, MO, USA). To visualize the bound antibody, we washed the cells and incubated them with 60 ng/mL of DAPI (Roche, Indianapolis, IN, USA) diluted in ethanol.

3. Alizarin Red Staining

To detect calcium deposits in hMSCs, induced cells were washed with phosphate-buffered saline (PBS) and fixed with 95% ethanol for 10 min, then washed five times with distilled water and stained with a 2% solution (pH 6.4) of Alizarin Red S (Sigma, St. Louis, MO, USA) for 5 min. Cells were washed for an additional 15 min to remove excess stain.

4. ALP Activity

Following osteogenic induction with or without sFRP-3 in 6 well-plates, 100 μL of water-soluble tetrazolium salt (WST)-8 reagent were added to each well containing 1 mL of Dulbecco's Modified Eagle's Medium and the plate was incubated for 1 h at 37 °C. The absorbance of each well was measured on a microplate reader (SmartSpeck 300; Bio-Rad, Hercules, CA, USA) at 450 nm. Each well was washed three times with PBS and incubated with 800 μL of *p*-nitrophenyl phosphate substrate solution (Sigma, St. Louis, MO, USA) for 1 h at 37 °C, and 600 μL of 0.2 N NaOH were added to each well to stop the reaction. The absorbance of each well was measured on a microplate reader at 415 nm. ALP activity was calculated by dividing the value by the number of cells calculated from the WST-8 assay.

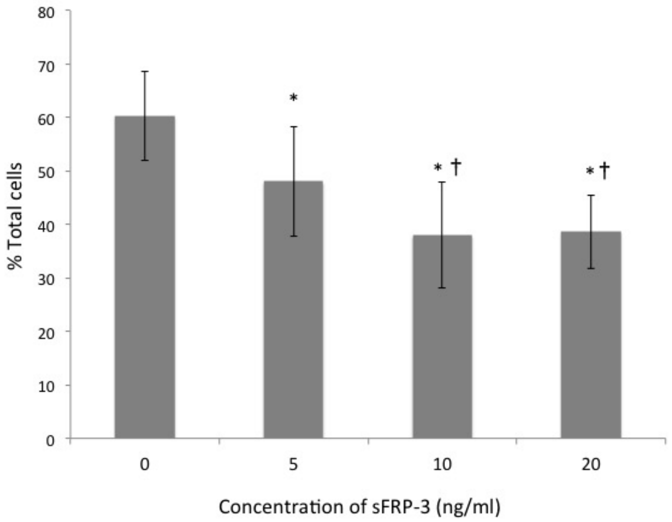

Figure S1. BrdU-positive cells were reduced with the addition of sFRP-3. hMSCs were cultured in MSGGM with several concentration of sFRP-3. In the 5, 10 and 20 ng/mL groups, BrdU-positive cells were statistically decreased compared with the group without sFRP-3 addition (0 ng/mL group) (* $p < 0.05$). In the 10 and 20 ng/mL groups, BrdU-positive cells were also statistically decreased compared with the 5 ng/mL group († $p < 0.05$). There was no statistical difference between 10 and 20 ng/mL groups.

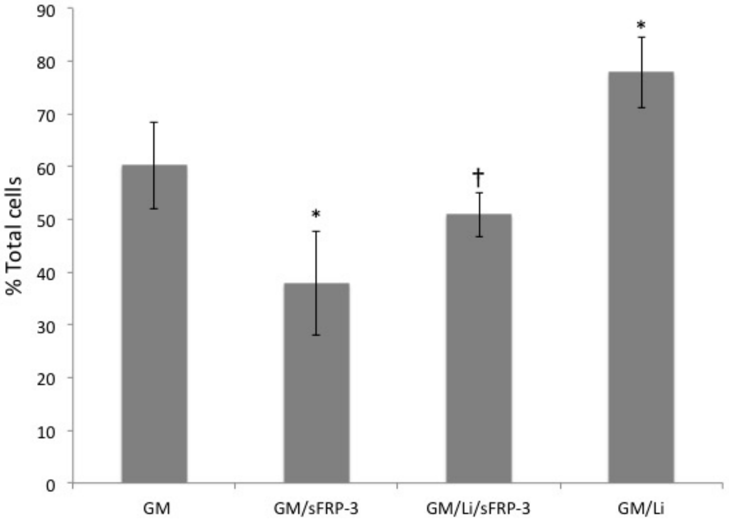

Figure S2. sFRP-3 reduced the number of BrdU-positive cells, which were improved with the addition of 4 mL of LiCl. BrdU-positive cells were statistically changed in GM/sFRP-3 and GM/LiCl groups compared with the GM group (* $p < 0.05$). Addition of LiCl (GM/Li/sFRP-3 group) statistically improved the number of BrdU-positive cells compared with GM/sFRP-3 group († $p < 0.05$). GM, MSCGM; Li, LiCl.

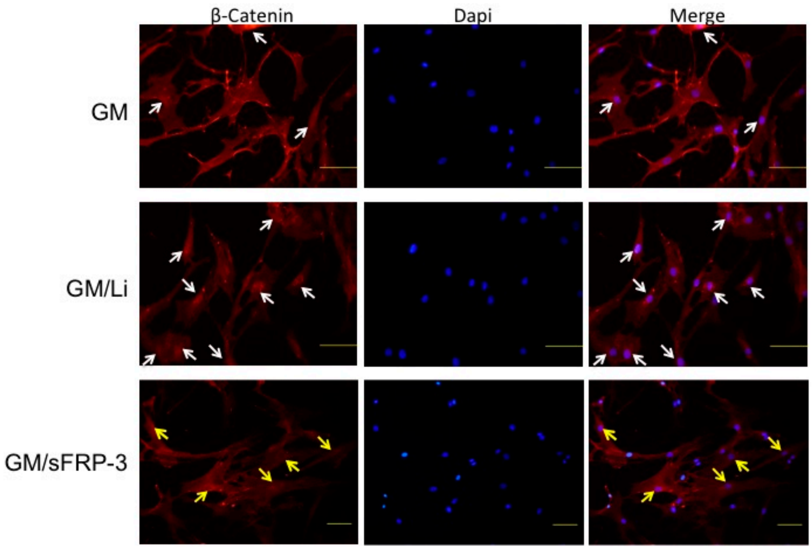

Figure S3. hMSCs were cultured in GM with addition of LiCl (4 mM; GM/Li) or sFRP-3 (10 ng/mL; GM/sFRP-3).White arrows indicate the nuclear localization of β-catenin and yellow arrows indicate the lack of nuclear localization of β-catenin. Scale bar, 200 μm; GM, MSCGM; Li, LiCl.

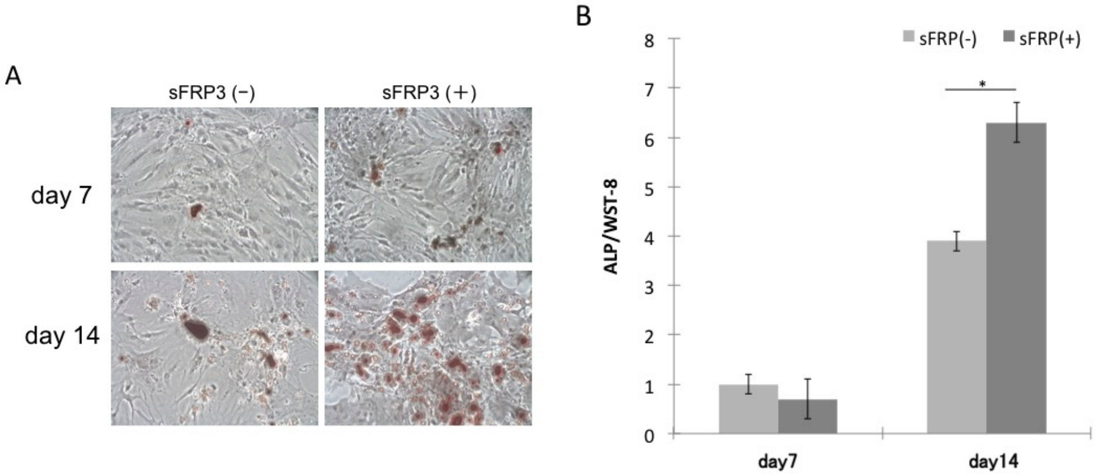

Figure S4. Effect of sFRP-3 treatment on calcification in hMCSs. (**A**) Representative Phase contrast micrographs of hMSCs cultured with or without sFRP-3 stained with Alizarin Red at indicated time points (40×); (**B**) Effect of sFRP-3 treatment on ALP activity in hMSCs. * $p < 0.05$.
